# Hip dysplasia—the Bernese contribution continues

**DOI:** 10.1093/jhps/hnae019

**Published:** 2024-07-25

**Authors:** Richard E Field

**Affiliations:** St George’s University of London, United Kingdom

March 2024 marked the 40th anniversary of the first Bernese periacetabular osteotomy (PAO) undertaken by Reinhold Ganz and Jeffrey Mast; on a 13-year-old girl with a proximal femoral focal deficiency and complex hip deformity [1]. It was a further 4 years before the first publication of this technique [2]. Over the past four decades, the superiority of the Bernese PAO over acetabular augmentation [3–5], single osteotomies [6–8], double osteotomies [9, 10] and triple osteotomies [11–13] has enabled tens of thousands of young people to enjoy active lives and delayed or prevented their need for hip arthroplasty.

Throughout our careers, we adapt and refine our practice. We adopt interventions that are demonstrated to provide superior outcomes in peer-reviewed publications, and we abandon interventions that have been superseded by better alternatives. However, it can take decades to demonstrate true superiority of one treatment over another and the best intervention for a particular individual may not be clear-cut. Selection of appropriate patients for PAO and the provision of advice on the potential requirement for further surgery are examples of such uncertainty. While every patient is unique, knowledge of trends in care can be helpful to guide our decision-making and the opinions that we provide.

In this issue, Stephen Bigach and the team at Northwestern University in Chicago, IL [14] provide a snapshot of the relative frequency that PAO is currently undertaken for men and women whose data can be accessed through the US PearlDiver database [15]. The authors provide a snapshot of the relative frequency that PAO is used for individuals of differing maturity, the incidence of such interventions per head of population, the frequency of interventions for associated intra-articular pathology or residual impingement, both concomitant with PAO and at a later date and the incidence of secondary interventions for removal of metalware. While the incidence of dysplasia requiring PAO surgery will vary in different geographic regions, this paper provides both PAO and non-PAO surgeons with information that will help them set patient expectations. It will also help PAO specialists to benchmark their own practices.

One of the questions that exercise patients considering PAO surgery is their prospect of returning to sporting activity. Patients want to know what level of activity they could anticipate undertaking and how quickly this could be achieved. PAO surgeons need to weigh up their prospects for achieving the desired outcome through isolated PAO surgery against the potential benefit of concomitant hip arthroscopy (HA) for intra-articular pathology or impingement. Also, the additional down-time if a second intervention becomes necessary to address residual symptomatic impingement. Phillip Wyatt and the team from the Virginia Commonwealth University in Richmond, Virginia [16] provide us with a systematic review demonstrating that there is a growing body of literature to answer these questions. However, they conclude that there is still a paucity of work that allows us to discern what combination of pre-operative dysplasia and intra-articular pathology is best served by combined PAO with concomitant HA. While there is no longer debate as to whether concomitant PAO and HA can be undertaken safely, it will be some years before the literature is sufficiently mature for surgeons to advise their patients who would be best served by isolated PAO and who should be listed for PAO with HA.

While acetabular dysplasia and its management have been extensively investigated over the past four decades, less attention has been paid to dysplasia of the proximal femur. Till Lerch and the team at the Inselspital, Bern University Hospital in Bern, have turned their attention to this problem and to the residual impingement that can occur after correction of decreased femoral anteversion. In their paper published in this issue [17], the Bernese team demonstrated that, as with changes in acetabular version, altering proximal femoral version can still leave residual impingement that requires concomitant correction. It is also noteworthy that the Bernese group favour plate fixation of their subtrochanteric osteotomies rather than the intramedullary nail technique that also has proponents [18]. It will be interesting to see which fixation technique gains the most support as the experience of this intervention grows over the next few decades.

## References

[1] Ganz R, Leunig M. Bernese periacetabular osteotomy (PAO): from its local inception to its worldwide adoption. *J Orthop Traumatol* 2023; 24: 55–73. 37917385 10.1186/s10195-023-00734-2 PMC10622391

[2] Ganz R, Klaue K, Vinh TS et al. A new periacetabular osteotomy for the treatment of hip dysplasias. *Clin Orthop Relat Res* 1988; 232: 29–36. 3383491

[3] Spitzy H . Artificial augmentation of the acetabular roof fixed with bony bolts. *Z Orthop Chir* 1923; 43: 284–94.

[4] Lance M . Bony shelf augmentation in dislocated and subluxated hips. *Presse Med* 1925; 33: 945–58.

[5] Chiari K . Results with ilium osteotomy for acetabular plasty. *Z Orthop* 1955; 87: 14–26. 13312490

[6] Salter RB . Innominate osteotomy in the treatment of congenital dislocation and subluxation of the hip. *J Bone Joint Surg B* 1961; 43: 518–39. 5921797

[7] Pemberton PA . Pericapsular osteotomy of the ilium for treatment of congenital subluxation and dislocation of the hip. *J Bone Joint Surg Am* 1965; 47: 65–86. 14256975

[8] Dega W . Anatomical and functional restitution in congenital hip dislocation by one-stage surgical procedure. *Arch Orthop Unfallchir* 1966; 60: 16–29. 5968884 10.1007/BF00415874

[9] Sutherland DH, Greenfield R. Double innominate osteotomy. *J Bone Joint Surg Am* 1977; 59A: 1082–91. 591540

[10] Hopf A . Acetabular relocation with double osteotomy for hip dysplasia and subluxation [in German]. *Z Orthop* 1966; 101: 559–86. 4230758

[11] Steel HH . Triple osteotomy of the inominate bone. *J Bone Joint Surg* 1973; 55A: 343–50. 4572223

[12] Tönnis D, Behrens K, Tscharani F. New triple osteotomy of the acetabulum to pivot the dysplastic acetabulum in adolescents and adults. *Z Orthop* 1981; 119: 253–65. 7269743 10.1055/s-2008-1051453

[13] Carlioz H, Khouri N, Hulin P. Triple juxta-acetabular osteotomy. *Rev Chir Orthop* 1982; 68: 497–501. 6220445

[14] Bigach SD, Thakkar AP, Buchler LT et al. Trends, demographics and reoperation rates of periacetabular osteotomy: an analysis from the PearlDiver database. *J Hip Preserv Surg* 2024; 11: 113–7. 10.1093/jhps/hnad040 PMC11272630 39070204

[15] The PearlDiver Mariner Patient Claims Database (PearlDiver Technologies, Colorado Springs, CO, USA) Available at: https://pearldiverinc.com Accessed: 15 May 2024.

[16] Wyatt P, Cole S, Satalich J et al. Periacetabular osteotomy with and without concomitant arthroscopy: a systematic review of evidence on post-operative activity levels and return to sport. *J Hip Preserv Surg* 2023; 11: 98–112. 10.1093/jhps/hnad043 PMC11272631 39070206

[17] Lerch TD, Meier MK, Hanke MS et al. Rotational femoral osteotomies and cam resection improve hip function and internal rotation for patients with anterior hip impingement and decreased femoral version. *J Hip Preserv Surg* 2023; 11: 85–91. 10.1093/jhps/hnad018 PMC11272641 39070203

[18] Buly RL, Sosa BR, Poultsides LA et al. Femoral derotation osteotomy in adults for version abnormalities. *J Am Acad Orthop Surg* 2018; 26: e416–25. 30106763 10.5435/JAAOS-D-17-00623 PMC6147096

